# Neutralizing Type I Interferon Autoantibodies in Japanese Patients With Severe COVID-19

**DOI:** 10.21203/rs.3.rs-1430985/v1

**Published:** 2022-03-11

**Authors:** Shohei Eto, Yoko Nukui, Miyuki Tsumura, Yu Nakagama, Kenichi Kashimada, Yoko Mizoguchi, Takanori Utsumi, Maki Taniguchi, Fumiaki Sakura, Kosuke Noma, Yusuke Yoshida, Shinichiro Ohshimo, Shintaro Nagashima, Keisuke Okamoto, Akifumi Endo, Kohsuke Imai, Hirokazu Kanegane, Hidenori Ohnishi, Shintaro Hirata, Eiji Sugiyama, Nobuaki Shime, Masanori Ito, Hiroki Ohge, Yasutoshi Kido, Paul Bastard, Jean-Laurent Casanova, Junko Tanaka, Tomohiro Morio, Satoshi Okada

**Affiliations:** Hiroshima University Graduate School of Biomedical and Health Science; Kyoto Prefectural University of Medicine; Hiroshima University Graduate School of Biomedical and Health Science; Osaka City University; Tokyo Medical and Dental University; Hiroshima University Graduate School of Biomedical and Health Science; Hiroshima University Graduate School of Biomedical and Health Science; Hiroshima University Graduate School of Biomedical and Health Science; Hiroshima University Graduate School of Biomedical and Health Science; Hiroshima University Graduate School of Biomedical and Health Science; Hiroshima University Hospital; Hiroshima University Graduate School of Biomedical and Health Science; Hiroshima University Graduate School of Biomedical and Health Sciences; Tokyo Medical and Dental University; Tokyo Medical and Dental University Hospital; Tokyo Medical and Dental University; Tokyo Medical and Dental University; Gifu University Graduate School of Medicine; Hiroshima University Hospital; Emeritus Professor of Hiroshima University; Hiroshima University Graduate School of Biomedical and Health Science; Hiroshima University Hospital; Hiroshima University Hospital; Osaka City University; University of Paris; Rockefeller Branch, The Rockefeller University; Hiroshima University Graduate School of Biomedical and Health Sciences; Tokyo Medical and Dental University; Hiroshima University Graduate School of Biomedical and Health Sciences

**Keywords:** COVID-19, Antibodies to type I IFNs, IFN-α2, IFN-ω, Neutralization assay, IFN-α2 concentration

## Abstract

**Purpose:**

Autoantibodies (aAbs) to type I interferons (IFNs) have been found in <1% of individuals under the age of 60 in the general population, with the prevalence increasing among those over 65. Neutralizing autoantibodies (naAbs) to type I IFNs have been found in at least 15% of patients with life-threatening COVID-19 pneumonia in several cohorts of primarily European descent. We aimed to define the prevalence of aAbs to IFN-α2 in 3,456 Japanese controls aged 20–91 and of aAbs and naAbs to IFN-α2 and IFN-ω in 627 Japanese COVID-19 patients aged 0–104, among whom were 170 critical, 235 severe, 112 moderate, 105 mild, and 5 asymptomatic infections.

**Methods:**

ELISA and ISRE reporter assays were used to detect aAbs and naAbs using E. coli-produced IFNs.

**Results:**

In an uninfected general Japanese population aged 20–91, we found aAbs in 0.087% of individuals. naAbs to type I IFNs (IFN-α2 and/or IFN-ω, 100 pg/mL) were detected in 10.6% of patients with critical infections, 2.6% of patients with severe infections, and ≤1% of patients with asymptomatic to mild infections. They were higher in COVID-19 patients over 50 (5.8%) than in younger patients (0%) and higher in men (5.5%) than in women (1.1%). A significant but not strong correlation between aAbs and naAbs to IFN-α2 existed (r=−0.307, p-value<0.0001), stressing the importance of measuring naAbs.

**Conclusion:**

In the largest study focusing on a single ethnic and geographic group, we show that Japanese individuals with pre-existing naAbs have a much higher risk of life-threatening COVID-19 pneumonia.

## Introduction

Coronavirus disease 2019 (COVID-19) is an infectious disease caused by severe acute respiratory syndrome coronavirus 2 (SARS-CoV-2). The clinical spectrum of COVID-19 varies in severity: approximately 80% of cases are asymptomatic, mild, or moderate (nonhypoxemic pneumonia), 15% of cases involve severe pneumonia (hypoxemia with O2<6 L/min), and 5% involve critical pneumonia (1). This virus is highly contagious and virulent, so the health care systems in endemic areas are in crisis. Therefore, appropriate triage of patients is needed before this infection becomes severe, and it is important to establish the risk factors that predict life-threatening COVID-19 pneumonia.

To date, the strong epidemiological risk factor for life-threatening COVID-19 is age (especially >65 years) (2–5). In contrast, other factors, such as male sex, cardiovascular disease, etc., are modestly associated with COVID-19 aggravation (6–8). However, there is important interindividual variability in the spectrum of COVID-19 severity, and in some patients, the disease becomes severe without these risk factors. In some of these cases, abnormalities of the innate immune system allowed viral replication in the early stage of the infection, and then excessive production of inflammatory cytokines triggered aggravation (9–14). Indeed, abnormalities in the type I interferon (IFN) signaling system have been reported to be strongly involved in COVID-19 aggravation. Zhang et al. reported that inborn errors of TLR3- and IRF7-dependent type I IFN induction and amplification could underlie 3.5% of life-threatening cases of COVID-19 pneumonia (15). Asano et al. reported that approximately 1.8% of male patients under the age of 60 years with critical COVID-19 pneumonia had X-linked recessive TLR7 deficiency (16). Even when these genes were not congenitally abnormal, the presence of neutralizing autoantibodies (naAbs) to type I IFNs can predate the infection and become a serious risk factor in COVID-19 aggravation. Bastard et al. reported that 10.2% of patients with life-threatening COVID-19 pneumonia had naAbs to type I IFNs, while 0.33% of healthy individuals and 0% of patients with asymptomatic/mild disease had them (17). Afterward, the authors revealed that 20% of both patients with critical COVID-19 over the age of 80 years and those with fatal COVID-19 of all ages had naAbs to type I IFNs (18). These findings suggest that approximately 5% of younger patients have a risk of aggravation due to genetic abnormalities associated with type I IFNs, and approximately 20% of older patients have a risk of aggravation due to naAbs to type I IFNs. The naAbs to type I IFNs have also been shown to underlie life-threatening adverse reactions to yellow fever vaccine (YFV) (19). However, the clinical symptoms associated with these naAbs are not apparent in other viral infections unless patients are infected with SARS-CoV-2. Therefore, it is difficult to predict the risk of aggravation due to naAbs to type I IFNs prior to SARS-CoV-2 infection. Although several subsequent studies supported these observations, accumulation of evidence is still required. It may be important to focus on a single ethnic group for precise characterization of the role of antibodies to type I IFNs in COVID-19 aggravation.

The current study aimed to clarify the prevalence of antibodies to type I IFNs in Japanese COVID-19 patients, estimating how much the presence of naAbs contribute as a risk factor in the Japanese population. This study also aimed to reveal the prevalence of aAbs in the uninfected Japanese population and discuss differences among ethnic groups compared to previous reports.

## Materials And Methods

### COVID-19 patients and individuals in the general population subjected to analysis

**Figure 1:**
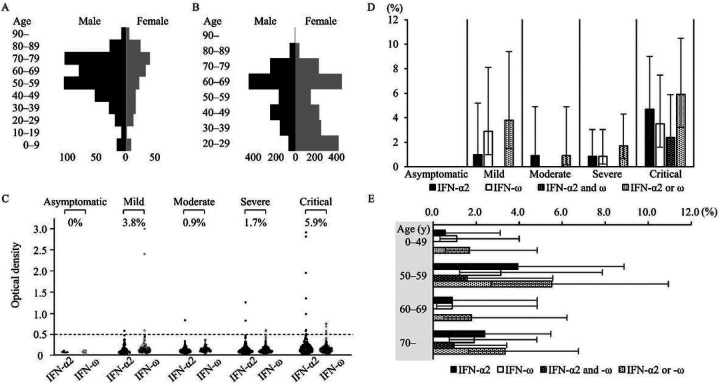
Characteristics of 627 patients with COVID-19 and 3,456 individuals from the general population and the results of aAbs to type I IFNs detected by ELISA in 627 patients with COVID-19. **A** Age and sex distribution of patients with COVID-19 (n=627). The median age of the COVID-19 patients was 61 years (IQR: 46–73 years); 70.2% were males, and 29.8% were females. **B** Age and sex distribution of individuals from the general population (n=3,456). The median age of subjects from the general population was 56 years (IQR: 37–67 years); 43.5% were males, and 56.5% were females. **C, D** Detection of aAbs to type I IFNs of patients with COVID-19 according to its severity with ELISA (n=627), including 170 critical, 235 severe, 112 moderate, 105 mild, and 5 asymptomatic infections. Their dot plot (C) and the prevalence (%) (D) are shown. The cutoff value of ELISA was 0.5 (O.D.). Antibodies against IFN-α2 and IFN-ω were measured as type I IFNs. **E** The prevalence of aAbs to type I IFNs of patients with COVID-19 according to age with ELISA. The prevalence of aAbs was high in patients with critical COVID-19 and in patients over 50 years old.

We conducted the study at Hiroshima University Hospital, Tokyo Medical and Dental University Medical Hospital, and Osaka City University Hospital. We enrolled 627 COVID-19 patients admitted to our institutes and 3,456 individuals from the general population (Fig. 1A, B, Table 1), which included 1,000 previously reported individuals (18). The detailed materials of the patients and the general population are described in the Supplemental materials and methods.

We assessed the severity of COVID-19 based on the Diagnosis and Treatment Protocol for Novel Coronavirus Pneumonia as previously described (17). A critical case was defined as a case involving mechanical ventilation (including intubation, high flow nasal cannula, continuous positive airway pressure, bilevel positive airway pressure, etc.), septic shock, any other organ failure and/or use of ECMO in the intensive care unit. Severe cases involved oxygen therapy < 6 L/min because of pneumonia. Moderate cases involved symptoms of mild pneumonia but no requirement for oxygen therapy. Mild cases were defined as some mild symptoms without pneumonia. Asymptomatic cases did not involve any symptoms.

### Neutralization assay of autoantibodies (aAbs) to type I IFNs

We performed luciferase reporter assays with reference to previous research (17). The detailed method of neutralization assay is described in the Supplemental materials and methods.

### Measurement of aAbs to type I IFNs and IFN-α2 concentration

The detailed methods of ELISA and ProQuantum^™^ Immunoassay are described in the Supplemental materials and methods.

### Statistical analysis

The detailed method of statistical analysis is described in the Supplemental materials and methods.

## Results

### The frequency of aAbs to type I IFNs was high in patients with critical COVID-19

We first measured aAbs to type I IFNs by ELISA in 627 Japanese COVID-19 patients aged 0–104 years, including 170 critical, 235 severe, 112 moderate, 105 mild, and 5 asymptomatic infections. We detected aAbs to IFN-α2 or IFN-ω at the following frequencies: 5.9% critical cases, 1.7% severe cases, 0.9% moderate cases, 3.8% mild cases, and 0% asymptomatic cases (Fig. 1C, Table S2). In detail, 4.7% (95% CI: 2.4–9.0) of patients with critical disease had aAbs to IFN-α2, 3.5% (95% CI: 1.6–7.5) to IFN-ω, and 2.4% (95% CI: 0.9–5.9) to both IFN-α2 and IFN-ω. The aAbs to IFN-α2 or IFN-ω were also detected in 1% or less of patients with asymptomatic to moderate disease and 3.8% of patients with mild disease. Unlike patients with critical COVID-19, none of the patients with asymptomatic to moderate disease had aAbs to both interferons (IFN-α2 and IFN-ω) (Fig. 1D, Tables 2, S2). Among patients over 50 years old, 3.6% (95% CI: 2.2–5.7) had aAbs to IFN-α2 or IFN-ω, while 1.7% (95% CI: 0.6–4.8) of patients younger than 50 years had these aAbs (Fig. 1E, Table 2). Overall, these aAbs to type I IFNs were detected more frequently in patients with critical disease and patients over 50 years old. However, the presence of isolated aAbs to IFN-α2 or IFN-ω was detected in some of the patients with asymptomatic to moderate disease in the current study.

### naAbs to type I IFNs were frequently detected in patients with critical COVID-19

**Figure 2:**
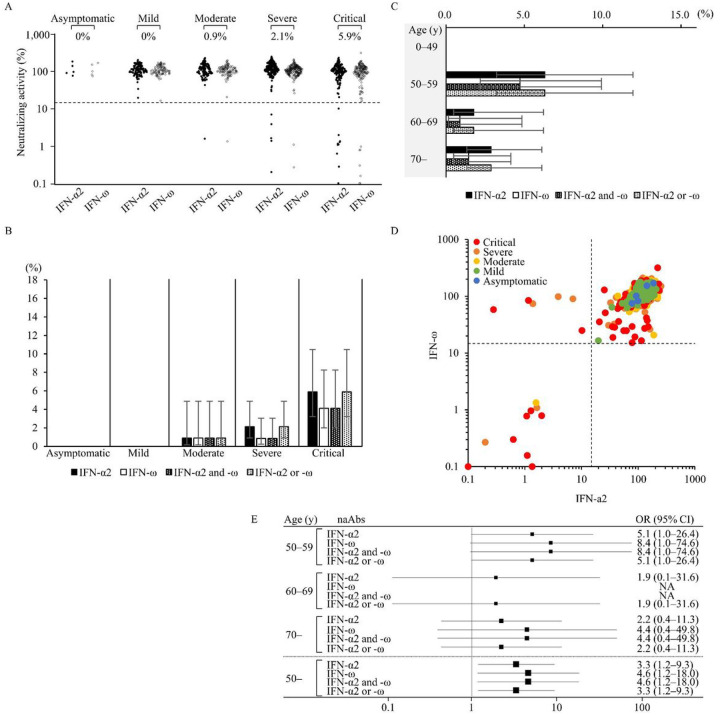
naAbs to type I IFNs detected by the neutralization assay in 627 patients with COVID-19 at a cytokine concentration of 10 ng/mL. Detection of naAbs to type I IFNs of patients with COVID-19 according to its severity with the neutralization assay (n=627). Antibodies against IFN-α2 and IFN-ω were measured as type I IFNs. The cutoff value of the neutralization assay was 15%. **A** Dot plot of the neutralization assay stimulated by 10 ng/mL of type I IFNs. The prevalence of naAbs was high in patients with critical COVID-19. **B** The prevalence of naAbs to type I IFNs of patients with COVID-19 according to its severity. The prevalence of naAbs was high in patients with critical COVID-19. **C** The prevalence of naAbs to type I IFNs in patients with COVID-19 according to age. The prevalence of naAbs was high in patients over 50 years old. **D** Neutralizing activity against type I IFNs was compared between IFN-α2 and IFN-ω stimulated by 10 ng/mL. All patients with neutralizing activity against IFN-ω had neutralizing activity against IFN-α2. **E** Odds ratio for critical COVID-19 relative to other milder severities. The odds ratio was high, especially for patients over 50 years old. NA denotes Not applicable.

ELISA detects aAbs that react with type I IFNs, but it does not always detect their neutralizing activity. We thus measured neutralizing activity against type I IFNs using the ISRE reporter assay in sera from 627 patients with COVID-19 (18). Sera were considered to have neutralizing activity if the induction of ISRE activity, which was normalized to Renilla luciferase activity, was less than 15% of the median values of healthy controls in reference to a previous report (18). The results of the neutralizing assay are summarized in Tables 3, S3 and S4. The prevalence of naAbs to high concentrations (10 ng/mL) of IFN-α2 or IFN-ω was found in 5.9% of critical cases, 2.1% of severe cases, 0.9% of moderate cases, 0% of mild cases, and 0% of asymptomatic cases (Fig. 2A, Table S3). In detail, 5.9% (95% CI: 3.2–10.5) of critical cases had naAbs to IFN-α2, 4.1% (95% CI: 2.0–8.3) of them to IFN-ω, and 4.1% (95% CI: 2.0–8.3) to both IFN-α2 and IFN-ω. On the other hand, less than 1% of patients with asymptomatic to moderate disease had naAbs to type I IFNs (Fig. 2B, table 3). Among patients over 50 years old, 3.6% (95% CI: 2.2–5.7) had naAbs to IFN-α2, 2.2% (95% CI: 1.2–4.1) to IFN-ω, and 2.2% (95% CI: 1.2–4.1) to both IFN-α2 and IFN-ω. In contrast, none of the patients younger than 50 years old had naAbs to type I IFNs (Fig. 2C, Table 3). Overall, 6 patients with critical/severe disease had naAbs to IFN-α2 alone, and 9 patients with critical/severe disease and only one with moderate disease had naAbs to both IFN-α2 and IFN-ω. On the other hand, we did not find patients with naAbs to IFN-ω alone in the 10 ng/mL condition (Fig. 2D, S1, Table S3). When we focused on COVID-19 patients over 50 years old, the odds ratio associated with COVID-19 aggravation among patients in critical condition was 3.3 (95% CI: 1.2–9.3) for the presence of naAbs to IFN-α2 alone, 4.6 (95% CI: 1.2–18.0) for both IFN-α2 and IFN-ω, and 4.6 (95% CI: 1.2–18.0) for IFN-ω alone (Fig. 2E).

**Figure 3:**
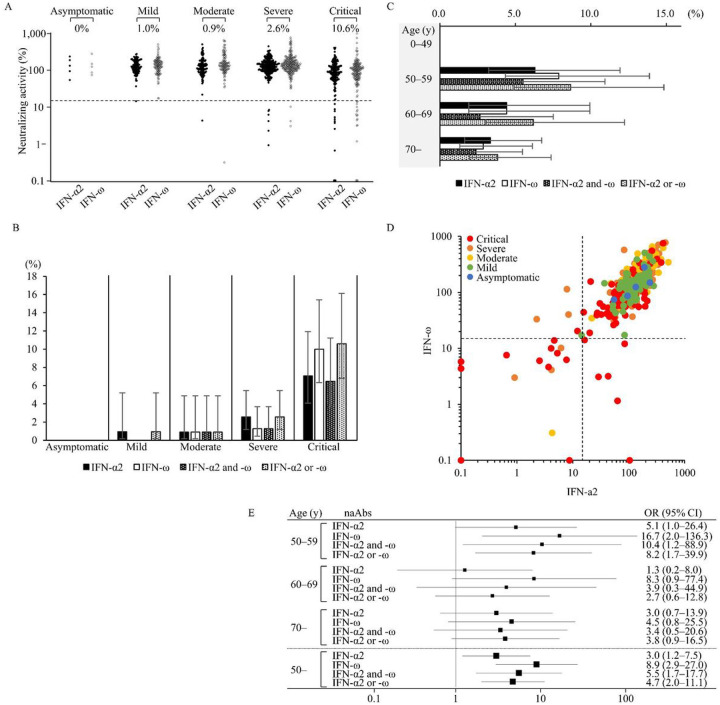
naAbs to type I IFNs detected by the neutralization assay in 627 patients with COVID-19 at a cytokine concentration of 100 pg/mL. Detection of naAbs to type I IFNs of patients with COVID-19 according to its severity with the neutralization assay (n=627). Antibodies against IFN-α2 and IFN-ω were measured as type I IFNs. The cutoff value of the neutralization assay was 15%. **A** Dot plot of the neutralization assay stimulated by 100 pg/mL of type I IFNs. The prevalence of naAbs was high in patients with critical COVID-19. **B** The prevalence of naAbs to type I IFNs of patients with COVID-19 according to its severity. The prevalence of naAbs was high in patients with critical COVID-19. **C** The prevalence of naAbs to type I IFNs in patients with COVID-19 according to age. The prevalence of naAbs was high in patients over 50 years old. **D** Neutralizing activity against type I IFNs was compared between IFN-α2 and IFN-ω stimulated by 100 pg/mL. **E** Odds ratio for critical COVID-19 relative to other milder severities. The odds ratio was high, especially for patients over 50 years old.

Next, we analyzed neutralizing activity in sera under more sensitive conditions by stimulating cells at lower concentrations (100 pg/mL) of IFN-α2 or IFN-ω. Under this condition, consistent with previous reports (18), the prevalence of naAbs was highly observed in 10.6% of critical cases, 2.6% of severe cases, 0.9% of moderate cases, 1.0% of mild cases, and 0% of asymptomatic cases (Fig. 3A, Table S3). In detail, 7.1% (95% CI: 4.1–11.9) of critical cases had naAbs to IFN-α2, 10.0% (95% CI: 6.3–15.4) to IFN-ω, and 6.5% (95% CI: 3.7–11.2) to both IFN-α2 and IFN-ω. Only 1% or less of the patients with asymptomatic to moderate disease had these naAbs to IFN-α2 or IFN-ω (Fig. 3B, Table 3). Among patients over 50 years old, 4.5% (95% CI: 2.9–6.8) had naAbs to IFN-α2, 4.7% (95% CI: 3.1–7.0) of them to IFN-ω, and 3.3% (95% CI: 2.0–5.4) to both IFN-α2 and IFN-ω. In contrast, none of the patients younger than 50 years old had naAbs to IFN-α2 or IFN-ω (Fig. 3C, Table 3). In this sensitive condition of the neutralizing assay, 4 patients with critical/severe disease had naAbs to IFN-α2, 6 patients with critical disease had naAbs to IFN-ω and 14 patients with critical/severe disease had naAbs to both IFN-α2 and IFN-ω. On the other hand, only one of the patients with mild disease had naAbs to IFN-α2, and only one of the patients with moderate disease had naAbs to both IFN-α2 and IFN-ω (Fig. 3D, S2, Table S3). Among COVID-19 patients over 50 years old, the odds ratio associated with COVID-19 aggravation among patients in critical condition was 3.0 (95% CI: 1.2–7.5) for the presence of naAbs to IFN-α2, 8.9 (95% CI: 2.9–27.0) for IFN-ω, and 5.5 (95% CI: 1.7–17.7) for both IFN-α2 and IFN-ω (Fig. 3E).

**Figure 4:**
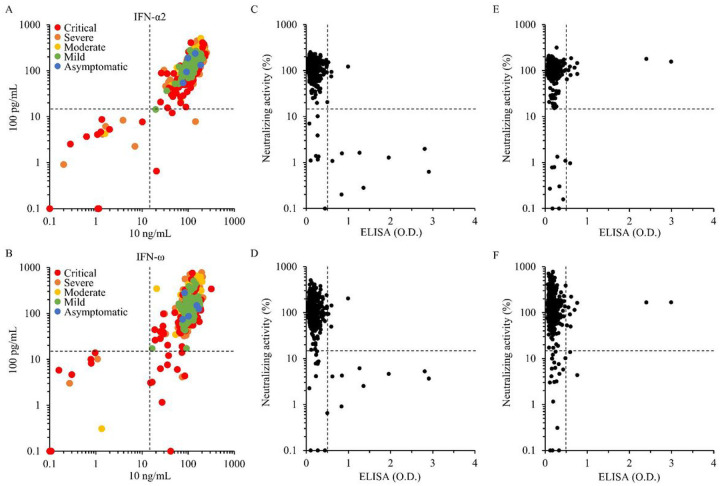
Comparison of neutralization assays between 10 ng/mL and 100 pg/mL and ELISA and neutralization assays of 627 patients with COVID-19. **A, B** Neutralizing activity against type I IFNs was compared between type I IFN concentrations of 100 pg/mL and 10 ng/mL stimulated by IFN-α2 (A) or IFN-ω (B). **C-F** aAbs to type I IFNs by ELISA were compared with naAbs by the neutralization assay at concentrations of 10 ng/mL IFN-α2 (C), 100 pg/mL IFN-α2 (D), 10 ng/mL IFN-ω (E) and 100 pg/mL IFN-ω (F). The cutoff value of ELISA was 0.5 (O.D.) and that of the neutralization assay was 15%.

When we focused on COVID-19 patients with naAbs against IFN-α2, neutralizing activity was detected in both the 10 ng/ml and 100 pg/mL conditions in 16 patients, while 4 other patients showed neutralizing activity against only 100 pg/ml IFN-α2. Among these 4 patients, 3 patients had critical/severe disease, and 1 patient had mild disease (Fig. 4A, S3). Regarding naAbs to IFN-ω, neutralizing activity was detected in both the 10 ng/mL and 100 pg/mL conditions in 10 patients, whereas 11 other patients showed neutralizing activity only against 100 pg/mL. Among these 11 patients, all patients had critical/severe disease (Fig. 4B, S4). It is known that the concentration of type I IFNs in the blood of patients with acute and benign SARS-CoV-2 infections ranges from 1 to 100 pg/mL (13, 20). Moreover, it has been experimentally proven that 100 pg/mL of type I IFNs can impair SARS-CoV-2 replication in epithelial cells. (18) Therefore, a neutralization assay using 100 pg/mL of type I IFNs, which reflects physiological conditions, detected naAbs more precisely than the assay using 10 ng/mL, especially naAbs to IFN-ω. The prevalence of naAbs by sex was 5.5% (95% CI: 3.7–8.0%) at 100 pg/mL and 3.4% (95% CI: 2.1–5.5%) at 10 ng/mL for males and 1.1% (95% CI: 0.3–3.8%) at 100 pg/mL and 0.5% (95% CI: 0.1–3.0%) at 10 ng/mL for females (Fisher’s exact test P value 0.0086 [100 pg/mL], 0.0488[10 ng/mL]) (Fig. S5, Table S4).

Overall, these naAbs to type I IFNs were detected more frequently in patients with critical disease, patients over 50 years old and men. These results fit with previous reports that identified a high prevalence, 10.2–18% in patients with critical disease, of naAbs to type I IFNs (table S5) (17, 18, 21–27).

### Comparison of the results of the neutralization assay and ELISA.

The neutralization assay can be considered a gold standard in assessing the biological effect of aAbs on type I IFNs. The ISRE reporter assay, especially that involving low concentrations of type I IFNs, gave us a sensitive test to assess naAbs to type I IFNs in COVID-19 patients. However, this assay is not suitable for assessing multiple samples because it requires gene introduction and takes 3 days to complete the whole process. On the other hand, ELISA-based assays enable us to analyze multiple samples with shorter experimental times. We thus compared the results of neutralizing activity against type I IFNs measured by the ISRE reporter assay with the results of aAbs to type I IFNs measured by ELISA. When the presence of naAbs to IFN-α2 was predicted by the results of aAbs to IFN-α2, the sensitivity was 50%, the specificity was 99.3%, the positive predictive value (PPV) was 66.7%, the negative predictive value (NPV) was 98.7% at 10 ng/mL (Fig. 4C), and these two detection methods had a weak negative correlation (a correlation coefficient −0.307 (95% CI: −0.376 −0.234, P value <0.0001)). For the 100 pg/mL condition, the sensitivity was 40%, the specificity was 99.3% (PPV of 66.7% and NPV of 98.0%), and these two detection methods had a weak negative correlation (a correlation coefficient −0.199 [95% CI: −0.273 −0.123, P value <0.0001]) (Fig. 4D). We thus realized that ELISA-based detection of aAbs to IFN-α2 can be an alternative method to enable testing of multiple samples, e.g., screening tests for the general population, and to evaluate antibodies to type I IFNs in sera. In contrast, for IFN-ω, ELISA failed to adequately detect the presence of naAbs to IFN-ω. Indeed, ELISA-based detection of aAbs to IFN-ω pointed out the presence of naAbs to IFN-ω (10 ng/mL condition) with a sensitivity of 10% and specificity of 98.4% (PPV of 9.1% and NPV of 98.5%) (Fig. 4E). Regarding the 100 pg/mL condition, aAbs to IFN-ω only indicated naAbs to IFN-ω with a sensitivity of 9.5% and a specificity of 98.5% (PPV of 18.2% and NPV of 96.9%) (Fig. 4F).

### Sera from COVID-19 patients with naAbs to IFN-α2 show low concentrations of IFN-α2.

**Figure 5:**
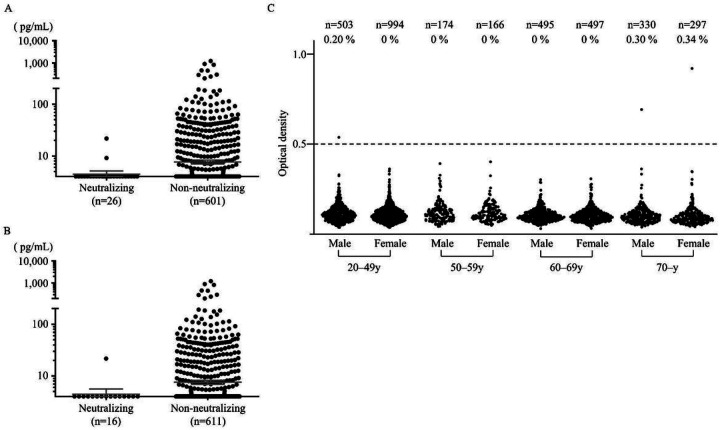
IFN-α2 concentration of patients with COVID-19 and prevalence of aAbs to IFN-α2 in 3,456 individuals in the general population. The IFN-α2 concentration in most of the patients with naAbs to IFN-α2 and/or IFN-ω was below the limit of quantification (<4 pg/mL). **A** Patients with naAbs to 100 pg/mL of IFN-α2 and/or IFN-ω (n=26) and patients without naAbs (n=601) were compared. **B** Patients with naAbs to 10 ng/mL of IFN-α2 and/or IFN-ω (n=16) and patients without naAbs (n=611) were compared. **C** aAbs to IFN-α2 in the general population were detected using ELISA. Overall, the prevalence of aAbs to IFN-α2 was 0.087% in 3,456 individuals.

We next measured the IFN-α2 concentration in sera from the 627 COVID-19 patients with the ProQuantum^™^ Human IFN alfa Immunoassay, which is a qPCR-based technique. The level of IFN-α2 in sera with naAbs to IFN-α2 and/or IFN-ω was significantly lower. In detail, 24 of 26 serum samples (neutralized at 100 pg/mL) and 15 of 16 serum samples (10 ng/mL) were below their detection limit (< 4 pg/mL). The concentrations of IFN-α2 between sera with and without naAbs were significantly different (Wilcoxon rank sum test, 100 pg/mL: p=0.0038, 10 ng/mL: 0.0086) (Fig. 5A, B). These results were consistent with a previous report (17).

### Prevalence of aAbs to IFN-α2 in uninfected individuals from the general Japanese population.

Measuring neutralizing activity against type I IFNs was ideal for predicting the risk of COVID-19 aggravation associated with the presence of aAbs to type I IFNs. However, neutralization assays using the ISRE reporter assay are not suitable for testing multiple samples. In the current study, ELISA-based prediction of naAbs to IFN-α2, at least in the 10 ng/mL condition, was thought to be trustworthy with a sensitivity of 50% and a specificity of 99.3%. Therefore, we estimated the prevalence of naAbs to type I IFNs in the Japanese population by detecting aAbs to IFN-α2 by ELISA. We measured aAbs to IFN-α2 in 3,456 Japanese individuals aged 20–91 years and unaffected by COVID-19. In this population, 3 individuals had aAbs to IFN-α2 (0.087% [95% CI: 0.0295–0.255%]) (Fig. 5C). These 3 individuals consisted of an 86-year-old female, a 78-year-old male, and a 42-year-old male.

## Discussion

The current study investigated aAbs and naAbs to type I IFNs in 627 patients with COVID-19 before the Delta variant became predominant. This is the second largest study on the scale of the samples, the largest study focusing on a single ethnic group, and the first in Asia. To minimize selection bias, we collected sera from COVID-19 patients from three geographically different areas (Tokyo, Osaka and Hiroshima) in Japan. The prevalence of naAbs to type I IFNs was high among patients with critical disease, elderly patients, and male COVID-19 patients. These observations were consistent with a previous study (17), providing strong evidence to support the risk of COVID-19 aggravation in individuals with naAbs to type I IFNs. The modest risk factors that are well known thus far are male sex (OR = 1.457) (7), cardiovascular disease (adjusted risk = 2.6) (6), chronic pulmonary disease (OR = 1.089) (7), diabetes mellitus with chronic complications (rate ratio = 1.295) (7). In contrast, the risk of COVID-19 aggravation among individuals with naAbs to type I IFNs was estimated to be higher (100 pg/mL of IFN-α2 and IFN-ω OR = 5.5, IFN-α2 or IFN-ω OR = 4.7, among patients over 50 years) than those with these modest risk factors. As shown in this study and a previous study (3, 5, 18), the prevalence of naAbs to type I IFNs is increased with older age and increases throughout life, which might be one of the reasons why age is the most striking epidemiological risk factor. On the other hand, naAbs to type I IFNs were also identified in 1% or less of patients with mild to moderate COVID-19. Therefore, although the presence of naAbs to type I IFNs is a strong risk factor for aggravation, a small portion of patients with these naAbs can have mild symptoms. This was also suggested by another case report (28).

It has been reported that only approximately 1% of the patients with naAbs to type I IFNs have naAbs to IFN-β (18). Therefore, IFN-β therapy might be one of the beneficial effects in cases with naAbs to type I IFNs (29–32). In addition, the removal of naAbs to type I IFNs with plasma exchange may be beneficial in the treatment of COVID-19 patients (33). Since these treatments may be effective only in the early stage of the infection (21), rapid tests to evaluate naAbs to type I IFNs are necessary for appropriate therapeutic interventions. The neutralization assay of aAbs to type I IFNs is the gold standard in assessing functional autoantibodies; however, it is not practical to use for rapid testing or for multiple samples due to its complexity. We thus compared the results of ELISA and the neutralization assay using the ISRE reporter assay and identified a certain correlation between aAbs and naAbs for IFN-α2. On the other hand, no correlation was observed for IFN-ω. A previous study pointed out a strong association between the severity of COVID-19 and naAbs to IFN-α2; in contrast, the risk of aggravation by naAbs to IFN-ω alone was not clear (18). We thus performed a systematic study by ELISA in 3,456 individuals without COVID-19 and revealed that 0.087% of them were positive for aAbs to IFN-α2. Since aAbs to IFN-α2 predicted the presence of naAbs to IFN-α2 with sensitivities of 50% (10 ng/dL condition) and 40% (100 pg/mL condition), the prevalence of naAbs to IFN-α2 was assumed to be 0.17–0.22%. This prevalence in the general population in Japan was slightly lower than that in a previous international study (0.33%) (17). In agreement that the prevalence of naAbs in patients with critical disease in Japan (10.6%) was lower than that (13.6%) in a previous international study, the prevalence of naAbs to type I IFNs might be slightly lower, or at least equivalent, in the Japanese population.

One of the limitations of this study in the general population was that the prevalence of naAbs to type I IFNs was estimated based on the results of ELISA. To accurately estimate the prevalence, the development of a simple method for assessing neutralizing activity that enables multisample processing is required. The current neutralization assay using the ISRE reporter assay requires gene transfection for each assay. On the other hand, the establishment of a stable cell line expressing the ISRE reporter gene may partially overcome this problem. In our study, some patients did not exhibit neutralizing activity against type I IFNs even if they had a high titer of aAbs to type I IFNs. This observation was also reported in another study (27) and was thought to be a false positive. This was partly because the detected aAbs can bind to target cytokines without neutralizing activity. The other possible explanation is that these aAbs may have neutralizing activity at concentrations lower than 100 pg/mL of stimulation. The neutralizing assay in this current study used 10% sera, so naAbs to 100 pg/mL of cytokines could neutralize 1000 pg/mL of cytokines. The IFN-α2 concentrations of most patients in this study were below 100 pg/mL in sera. Therefore, the neutralization assay using 10 pg/mL cytokine stimulation might be worth trying. These possibilities are thought to be topics for future analysis. Despite having some limitations, this was the first study to characterize the relationship between naAbs to type I IFNs and COVID-19 aggravation in a Japanese population and the second largest study on this theme, providing strong evidence to support the contribution of naAbs to type I IFNs to the risk of COVID-19 aggravation.

## Supplementary Material

Supplement 1

## Figures and Tables

**Table 1.** Characteristics of 627 patients with COVID-19 and 3,456 general population in this study

	627 patients with COVTD-19			3,456 general population in this study		
Age (years)	Total cases [n = 627](%)	Male [n = 440]	Female [n = 187]	Total cases [n = 3,456](%)	Male [n = 1,502]	Female [n = 1,954]
0–9	24 (3.8%)	15	9	-	-	-
10–19	9 (1.4%)	7	2	-	-	-
20–29	31 (4.9%)	18	13	536 (15.5%)	72	464
30–39	45 (7.2%)	28	17	439 (12.7%)	164	275
40–49	69 (11.0%)	52	17	522 (15.1%)	267	255
50–59	127 (20.3%)	104	23	340 (9.8%)	174	166
60–69	113 (18.0%)	79	34	992 (28.7%)	495	497
70–79	144 (23.0%)	103	41	519 (15.0%)	267	252
80–89	52 (8.3%)	27	25	105 (3.0%)	60	45
90–	13 (2.1%)	7	6	3 (0.1%)	3	0
Severity	Total cases [n = 627](%)	Male [n = 440]	Female [n = 187]			
Asymptomatic	5 (0.8%)	1	4			
Mild	105 (16.7%)	67	38			
Moderate	112 (17.9%)	68	44			
Severe	235 (37.5%)	166	69			
Critical	170 (27.1%)	138	32			

**Table 2.** aAbs to type I IFNs in 627 patients with COVID-19

aAbs detected by EL SIA					
Severity	IFN-α2	IFN-ω	IFN-α2 and -ω	IFN-α2 or -ω	No. of patients in this severity
Asymptomatic	0 (0.0%)	0 (0.0%)	0 (0.0%)	0 (0.0%)	5
Mild	1 (1.0%[0.2–5.2])	3 (2.9%[1.0–8.1])	0 (0.0%)	4 (3.8%[1.5–9.4])	105
Moderate	1 (0.9%[0.2–4.9])	0 (0.0%)	0 (0.0%)	1 (0.9% [0.2–4.9])	112
Severe	2 (0.9%[0.2–3.1])	2 (0.9%[0.2–3.1])	0 (0.0%)	4 (1.7%[0.7–4.3])	235
Critical	8 (4.7%[2.4–9.0])	6 (3.5%[1.6–7.5])	4 (2.4%[0.9–5.9])	10 (5.9%[3.2–10.5])	170
Total	12	11	4	19	627
Age (years)	IFN-α2	IFN-ω	IFN-α2 and -ω	IFN-α2 or -ω	No. of patients in this severity
0–49	1 (0.6%[0.1–3.1])	2 (1.1%[0.3–4.0])	0 (0.0%)	3 (1.7%[0.6–4.8])	178
**50–**	**11** **(25%[1.4–4.3])**	**9** **(2.0%[1.1–3.8])**	**4** **(0.9%[0.3–2.3])**	**16** **(3.6%[2.2–5.7])**	**449**
50–59	5 (3.9%[1.7–8.9])	4 (3.2%[1.2–7.9])	2 (1.6%[0.4–5.6])	7 (5.5%[2.7–10.9])	127
60–69	1 (0.9%[0.2–4.8])	1 (0.9%[0.2–4.8])	0 (0.0%)	2 (1.8%[0.5–6.2])	113
70–	5 (2.4%[1.0–5.5])	4 (1.9%[0.7–4.8])	2 (1.0%[0.3–3.4])	7 (3.3%[1.6–6.8])	209

**Table 3.** naAbs to type I IFNs in 627 patients with COVID-19

nabs detected by Neutralization assay									
	10 ng/mL				100 pg/mL				
Severity	IFN-α2	IFN-ω	IFN-α2 and -ω	IFN-α2 or -ω	IFN-α2	IFN-ω	IFN-α2 and -ω	IFN-α2 or -ω	No. of patients in this severity
Asymptomatic	0 (0.0%)	0 (0.0%)	0 (0.0%)	0 (0.0%)	0 (0.0%)	0 (0.0%)	0 (0.0%)	0 (0.0%)	5
Mild	0 (0.0%)	0 (0.0%)	0 (0.0%)	0 (0.0%)	1 (1.0%[0.2–5.2])	0 (0.0%)	0 (0.0%)	1 (1.0%[0.2–5.2])	105
Moderate	1 (0.9%[0.2–4.9])	1 (0.9%[0.2–4.9])	1 (0.9%[0.2–4.9])	l (0.9%[0.2–4.9])	1 (0.9%[0.2–4.9])	1 (0.9%[0.2–4.9])	1 (0.9%[0.2–4.9])	1 (0.9%[0.2–4.9])	112
Severs	5 (2.1%[0.9–4.9])	2 (0.9%[0.2–3.0])	2 (0.9%[0.2–3.0])	5 (2.1%[0.9–4.9])	6 (2.6%[1.2–5.5])	3 (1.3%[0.4–3.7])	3 (1.3%[0.4–3.7])	6 (2.6%[1.2–5.5])	235
Critical	10 (5.9%[3.2–10.5])	7 (4.1%[2.0–8.3])	7 (4.1%[2.0–8.3])	10 5.9%[3.2–10.5])	12 (7.1%[4.1–11.9])	17 (10.0%[6.3–15.4])	11 (6.5%[3.7–11.2])	18 (10.6%[6.8–16.1])	170
Total	6	0	10	16	5	6	15	26	627
Age(years)	IFN-α2	IFN-ω	IFN-α2 and -ω	IFN-α2 or -ω	IFN-α2	IFN-ω	IFN-α2 and -ω	IFN-α2 or -ω	No. of patients in this severity
0–49	0 (0.0%)	0 (0.0%)	0 (0.0%))	0 (0.0%)	0 (0.0%)	0 (0.0%)	0 (0.0%)	0 (0.0%)	178
**50–**	**16** **(3.6%[2.2–5.7])**	**10** **(2.2%[1.2–4.1])**	**10** **(2.2%[1.2–4.1])**	**16** **(3.6%[2.2–5.7])**	**20** **(4.5%[2.9–6.8])**	**21** **(4.7%[3.1–7.0])**	**15** **(3.3%[2.0–5.4])**	**26** **(5.8%[4.0–8.3])**	**449**
50–59	8 (6.3%[3.2–11.9])	6 (4.7%[2.2–9.9])	6 (4.7%[2.2–9.9])	8 (6.3%[3.2–11.9])	8 (6.3%[3.2–11.9])	10 (7.9%[4.3–13.9])	7 (5.5%[2.7–10.9])	11 (8.7%[4.9–14.8])	127
60–69	2 (1.8%[0.5–6.2])	1 (0.9%[0.2–4.8])	1 (0.9%[0.2–4.8])	2 (1.8%[0.5–6.2])	5 (4.4%[1.9–9.9])	5 (4.4%[1.9–9.9])	3 (2.7%[0.9–7.5%])	7 (6.2%[3.0–12.2])	113
70–	6 (2.9%[1.3–6.1])	3 (1.4%[0.5–4.1])	3 (1.4%[0.5–4.1])	6 (2.9%[1.3–6.1])	7 (3.3%[1.6–6.8])	6 (2.9%[1.3–6.1])	5 (2.4%[1.0–5.5])	8 (3.8%[2.0–7.4])	209

## Data Availability

The datasets generated during and/or analyzed during the current study are available from the corresponding author on reasonable request.
