# Supplementary material for: Neutralizing Type I Interferon Autoantibodies in Japanese Patients With Severe COVID-19
Source: Res Sq. 2022 Mar 11:rs.3.rs-1430985. Preprint. [Version 1] doi: 10.21203/rs.3.rs-1430985/v1 (PMC8923117; doi:10.21203/rs.3.rs-1430985/v1)
Supplement: Supplement 1 [file 381e2857ae901431cbe7ee4e.docx]

**Electronic Supplementary Materials**

**Neutralizing type I interferon autoantibodies in Japanese patients with severe COVID-19**

**Authors**

Shohei Eto^1, @^, Yoko Nukui^2,3 @^, Miyuki Tsumura^1^, Yu Nakagama^4^, Kenichi Kashimada^5^, Yoko Mizoguchi^1^, Takanori Utsumi^1^, Maki Taniguchi^1^, Fumiaki Sakura^1^, Kosuke Noma^1^, Yusuke Yoshida^6^, Shinichiro Ohshimo^7^, Shintaro Nagashima^8^, Keisuke Okamoto^5^, Akifumi Endo^9^, Kohsuke Imai^10^, Hirokazu Kanegane^11^, Hidenori Ohnishi^12^, Shintaro Hirata^6^, Eiji Sugiyama^13^, Nobuaki Shime^7^, Masanori Ito^14^, Hiroki Ohge^15^, Yasutoshi Kido^4^, Paul Bastard^16-18^, Jean-Laurent Casanova^16-19^, Junko Tanaka^8^, Tomohiro Morio^5^, Satoshi Okada^1^

**Institutions**

^1^Department of Pediatrics, Hiroshima University Graduate School of Biomedical and Health Science, Hiroshima, Japan.

^2^Department of Infection Control and Prevention, Tokyo Medical and Dental University Hospital, Tokyo, Japan.

^3^Department of Infection Control and Laboratory Medicine, Kyoto Prefectural University of Medicine, Kyoto, Japan.

^4^Department of Parasitology, Graduate School of Medicine, Osaka City University, Osaka, Japan.

^5^Department of Pediatrics and Developmental Biology, Graduate School of Medical and Dental Sciences, Tokyo Medical and Dental University, Tokyo, Japan.

^6^Department of Clinical Immunology and Rheumatology, Hiroshima University Hospital, Hiroshima, Japan.

^7^Department of Emergency and Critical Care Medicine, Hiroshima University Graduate School of Biomedical and Health Science, Hiroshima, Japan.

^8^Department of Epidemiology, Infectious Disease Control and Prevention, Hiroshima University Graduate School of Biomedical and Health Sciences, Hiroshima, Japan.

^9^Clinical Research Center, Tokyo Medical and Dental University Hospital, Tokyo, Japan.

^10^Department of Community Pediatrics, Perinatal and Maternal Medicine, Graduate School of Medical and Dental Sciences, Tokyo Medical and Dental University, Tokyo, Japan.

^11^Department of Child Health and Development, Tokyo Medical and Dental University, Tokyo, Japan.

^12^Department of Pediatrics, Gifu University Graduate School of Medicine, Gifu, Japan.

^13^Emeritus Professor of Hiroshima University, Hiroshima, Japan.

^14^Department of General Internal Medicine, Hiroshima University Hospital, Hiroshima, Japan.

^15^Department of Infectious Diseases, Hiroshima University Hospital, Hiroshima, Japan.

^16^Laboratory of Human Genetics of Infectious Diseases, Necker Branch, INSERM U1163, Necker Hospital for Sick Children, Paris, France.

^17^University of Paris, Imagine Institute, Paris, France.

^18^St. Giles Laboratory of Human Genetics of Infectious Diseases, Rockefeller Branch, The Rockefeller University, New York, NY, USA.

^19^Howard Hughes Medical Institute, New York, NY, USA.

^@^ These authors contributed equally to this work

**Corresponding Author**

Satoshi Okada, MD, PhD

E-mail: sokada@hiroshima-u.ac.jp

**This file includes**

**Supplemental Tables 1, 2, 3, 4, 5**

**Supplemental Figures 1, 2, 3, 4, 5**

**Supplemental materials and methods**

**Table S1 Characteristics of general population before the appearance of COVID-19 and after the appearance of COVID-19**

**Table S2 aAbs to type I IFNs in 627 patients with COVID-19**

**Table S3 naAbs to type I IFNs in 627 patients with COVID-19**

**Table S4 The prevalence of naAbs to type I IFNs detected by the neutralization assay in 440 male and 187 female patients with COVID-19**

**Table S5 Summary of reported articles of antibodies to type I IFNs**

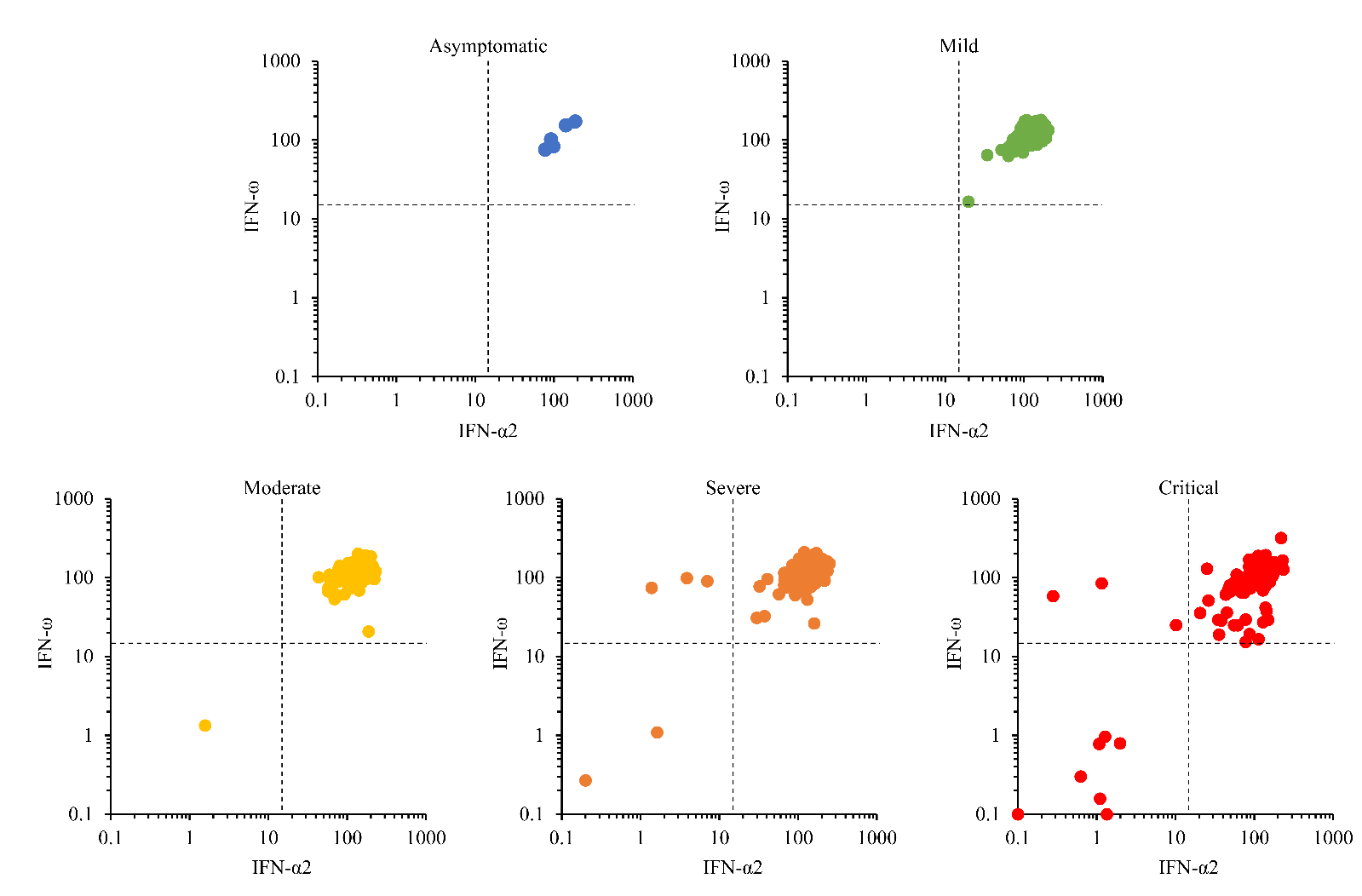


**Figure S1**

naAbs to type I IFNs in 627 patients with COVID-19 at a cytokine concentration of 10 ng/mL. Neutralizing activity against 10 ng/mL IFN-α2 or IFN-ω in patients with COVID-19 according to its severity (n=627). The cutoff value of neutralizing activity was 15%.


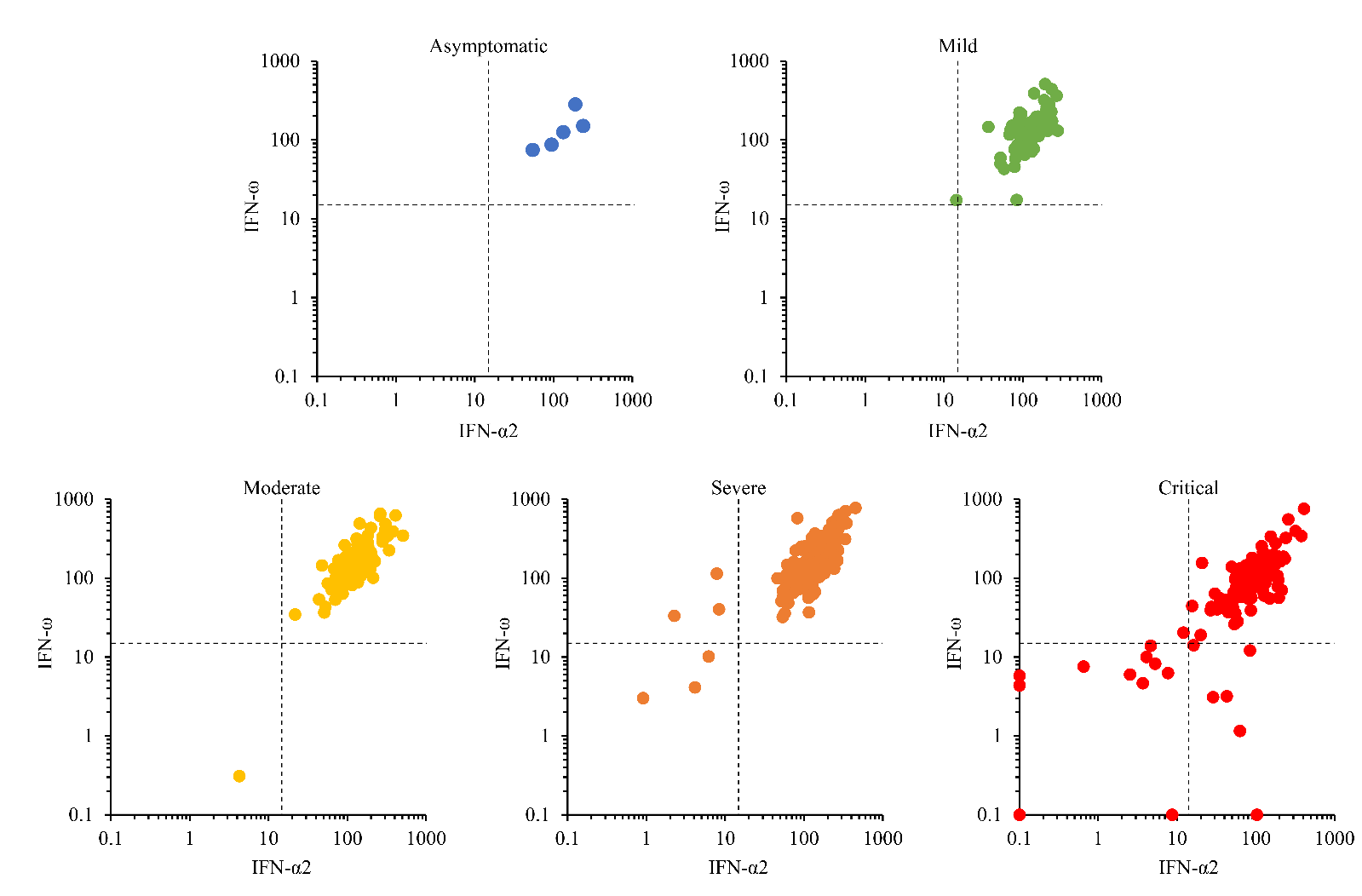


**Figure S2**

naAbs to type I IFNs in 627 patients with COVID-19 at a cytokine concentration of 100 pg/mL. Neutralizing activity against 100 pg/mL IFN-α2 or IFN-ω in patients with COVID-19 according to its severity (n=627). The cutoff value of neutralizing activity was 15%.


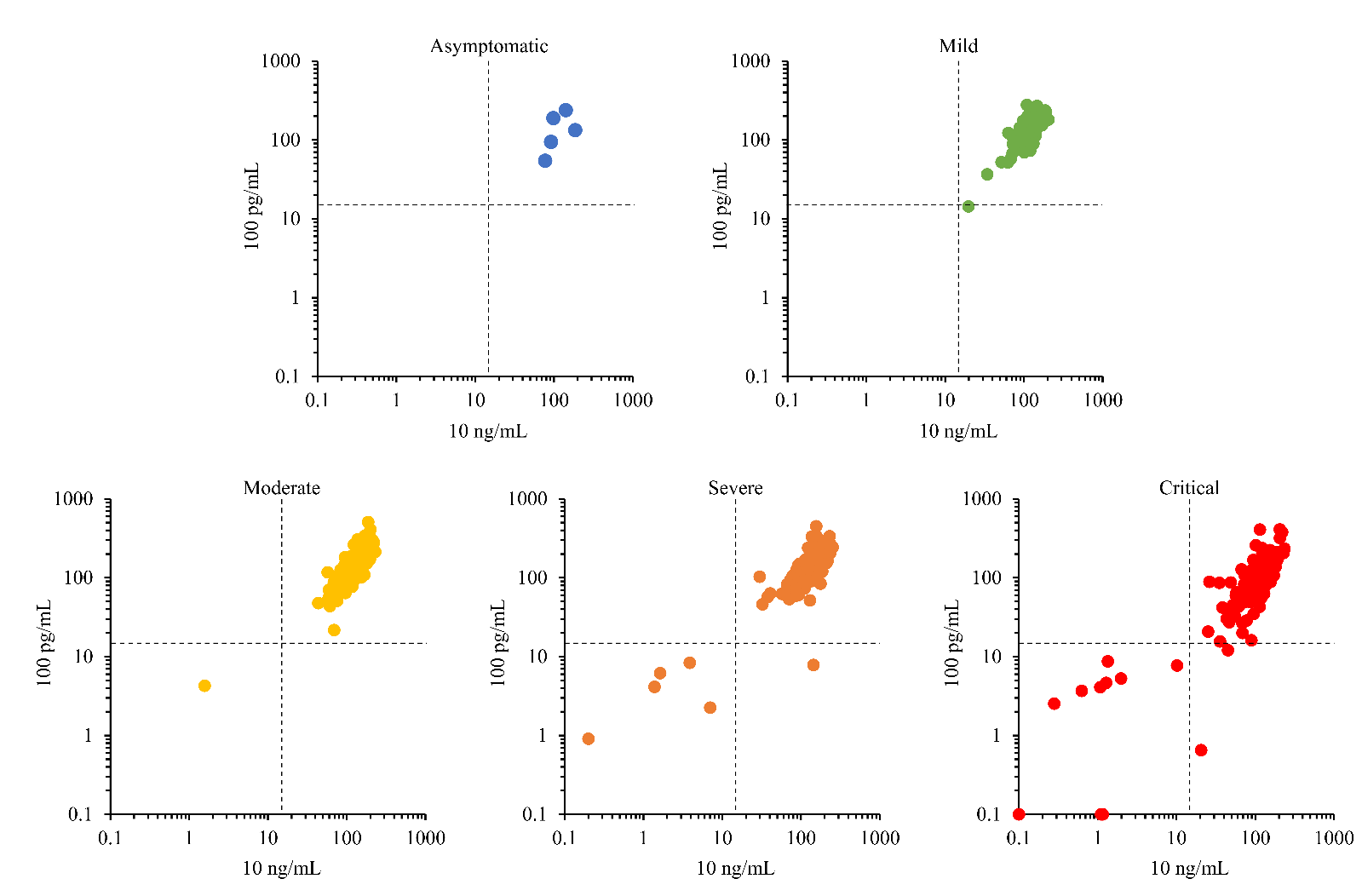


**Figure S3**

naAbs to IFN-α2 in 627 patients with COVID-19. Neutralizing activity against IFN-α2 in patients with COVID-19 according to its severity (n=627). The cutoff value of neutralizing activity was 15%. Activity levels of 10 ng/mL and 100 pg/mL were compared.


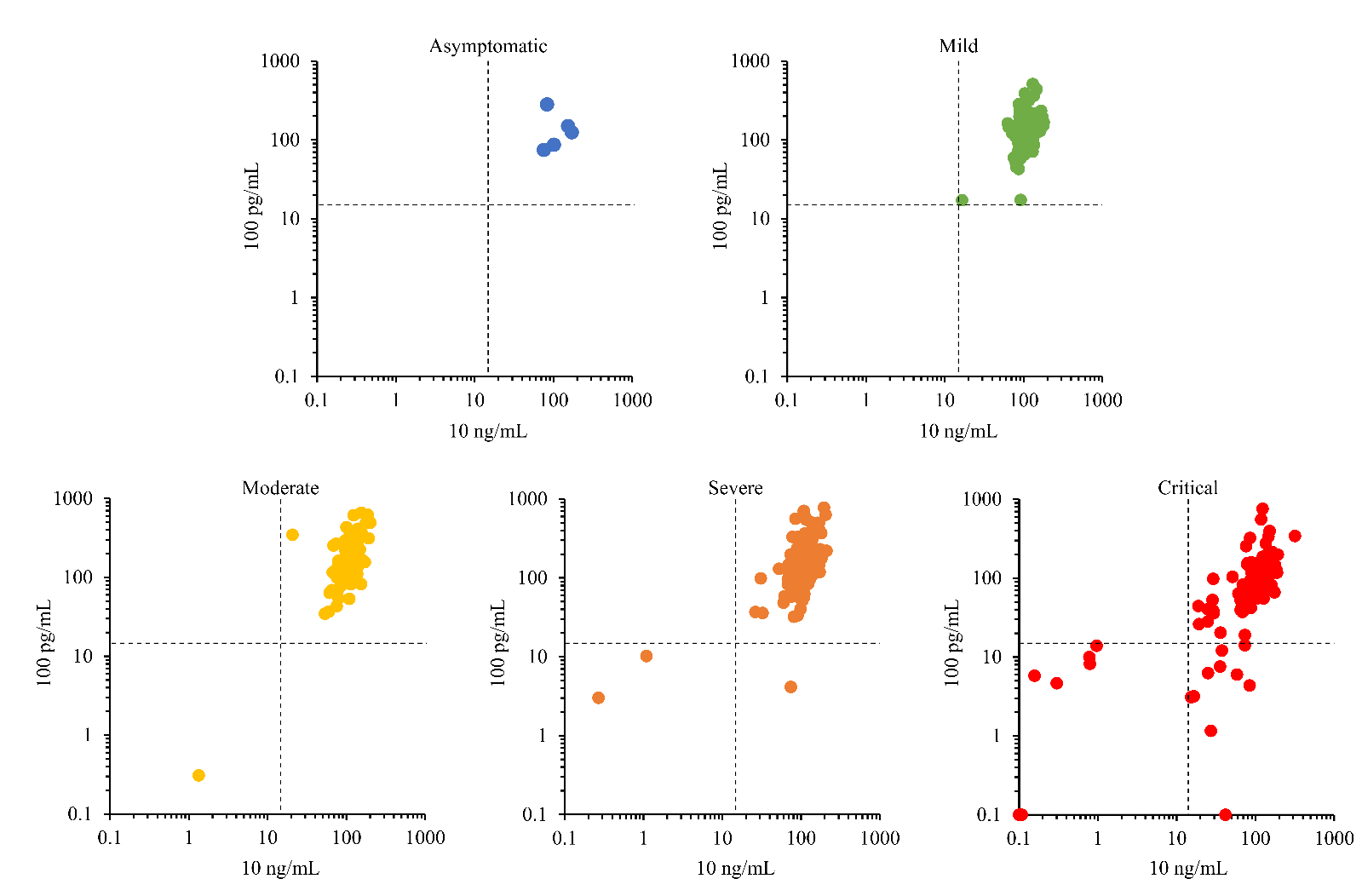


**Figure S4**

naAbs to IFN-ω in 627 patients with COVID-19. Neutralizing activity against IFN-ω in patients with COVID-19 according to its severity (n=627). The cutoff value of neutralizing activity was 15%. Activity levels of 10 ng/mL and 100 pg/mL were compared.


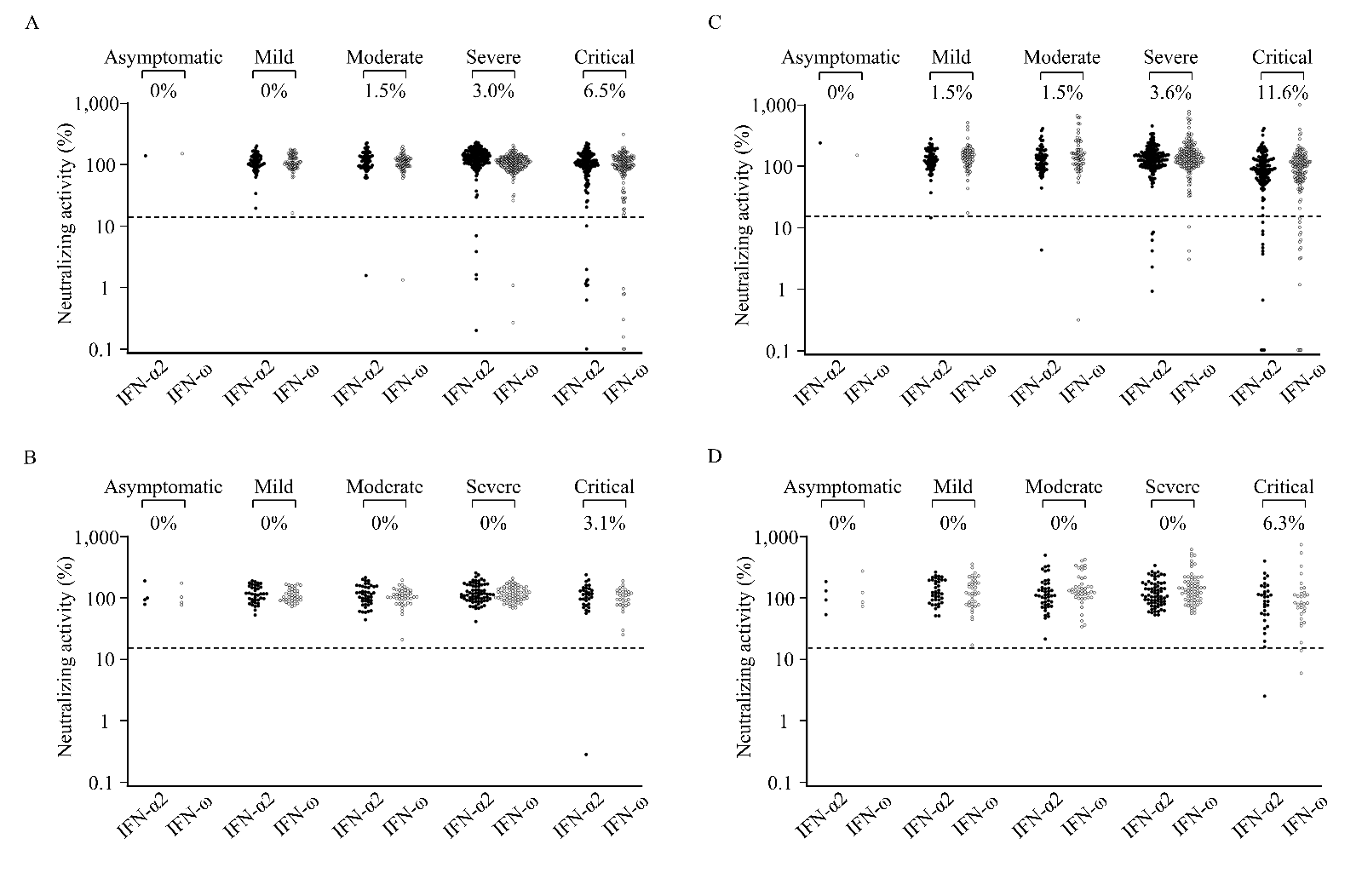


**Figure S5**

naAbs to type I IFNs in 627 patients with COVID-19 classified by sex and cytokine concentration. Neutralizing activity against IFN-α2 or IFN-ω in patients with COVID-19 according to its severity (n=627). 138 critical, 166 severe, 68 moderate, 67 mild, and 1 asymptomatic infections in male patients. 32 critical, 69 severe, 44 moderate, 38 mild, and 4 asymptomatic infections in female patients. The cutoff value of neutralizing activity was 15%. **A** The neutralization assay to 10 ng/mL of type I IFNs in males. **B** The neutralization assay against 10 ng/mL of type I IFNs in females. **C** The neutralization assay against 100 pg/mL of type I IFNs in males. **D** The neutralization assay to 100 pg/mL of type I IFNs in females.

**Supplemental materials and methods**

**COVID-19 patients and individuals in the general population subjected to analysis**

We conducted the study at Hiroshima University Hospital, Tokyo Medical and Dental University Medical Hospital, and Osaka City University Hospital. We enrolled 627 COVID-19 patients admitted to our institutes and 3,456 individuals from the general population, which included 1,000 previously reported individuals.^1^ The general population consisted of 2,069 people with annual medical check-ups from April 2017 through March 2018 (before the appearance of SARS-CoV-2) and 1,387 medical staff without a history of COVID-19 infection after the appearance of SARS-CoV-2. The median age of the COVID-19 patients was 61 years (IQR: 46-73 years); 70.2% were males, and 29.8% were females (Fig 1A, Table 1). The median age of the general population was 56 years (IQR: 37-67 years); 43.5% were males, and 56.5% were females (Fig 1B, Table 1, E1). All subjects were recruited according to ethics codes approved by the local institutional review boards. The diagnosis of COVID-19 was made by direct detection of SARS-CoV-2 RNA by nucleic acid amplification tests. These COVID-19 samples were collected by August 2021 before the appearance of the Delta variant.

**Neutralization assay of autoantibodies (aAbs) to type I IFNs**

We performed luciferase reporter assays with reference to previous research.^2^ We seeded 96-well plates at a cell density of 4.0×10^4^ cells/well in 100 μL media and incubated them overnight at 37°C. Cells were 70-80% confluent after 16 hours. We transfected HEK293T cells with a luciferase reporter plasmid vector containing the firefly luciferase gene driven by the promoter of the interferon-stimulated response element (ISRE) in the pGL4.45 backbone and a control reporter plasmid vector pRL-SV40 for normalization. We transfected these plasmids using X-tremeGene 9 transfection reagent (Roche Diagnostics, Basel, Switzerland) for 24 hours. We added 10% serum/plasma from individuals from the general population or patients diluted with Dulbecco’s Modified Eagle medium, (DMEM) (Thermo Fisher Scientific) supplemented with 2% HyClone^TM^ fetal bovine serum (GE Healthcare Life Sciences, IL, USA). We stimulated cells with rhIFN-α2 for 8 hours or rhIFN-ω for 12 hours at cytokine concentrations of 10 ng/mL or 100 pg/mL, respectively, at 37°C. Finally, we lysed the cells and measured the luciferase levels with a Dual-Luciferase Reporter assay system (Promega, WI, USA) and an EnSpire plate reader. We calculated the ratio of firefly luciferase luminescence to Renilla luciferase luminescence for each sample, and the ratio was subtracted by the median value of non-IFN-stimulated wells that contained sera of healthy controls. These ratios were expressed as percentages of the median value of healthy controls who did not have naAbs tested on the same day. The samples were considered to have neutralizing activity if these ratios were below 15%. We tested each sample once.

**Enzyme-linked immunosorbent assay (ELISA): Detection of aAbs to type I IFNs**

We performed ELISA with reference to previous research.^2^ We coated 96-well ELISA plates (F96 MaxiSorp Nunc-Immuno Plate; Thermo Fisher Scientific, MA, USA) overnight at 4 ℃ with 1 μg/mL rhIFN-α2 (Human IFN-a2a research grade, Miltenyi Biotec, CA, USA) at 100 μL/well and 1 μg/mL rhIFN-ω (human IFN-ω, eBioscience, CA, USA) at 100 μL/well. We washed the plates with PBS three times and blocked the plates with blocking medium (PBS with 5% nonfat milk powder) for 1 hour at room temperature on an agitator. Then, we washed plates with PBS containing 0.005% Tween and added 100 μL of 1/50 plasma dilutions (High Performance ELISA buffer, MA, USA) for 2 hours at room temperature on an agitator. Next, we washed the plates with PBS containing 0.005% Tween, added 2 μg/mL secondary antibody (goat anti-human IgG IgA IgM (Fc specific) conjugated with horseradish peroxidase, Nordic MUbio, Susteren, Netherlands) at 100 μL/well and incubated the plates for 1 hour at room temperature on an agitator while protected from light. Finally, we washed the plates with PBS containing 0.005% Tween, added 100 μL/well substrate (KPL SureBlue^TM^ TMB Microwell Peroxidase Substrate, MA, USA), kept the plates on an agitator for 5 minutes, added the same amount of 1.8 M H_2_SO_4_, and measured the optical density (450 nm/630 nm) with an EnSpire plate reader (PerkinElmer, MA, USA).

We used a machine (Wellwash^TM^ Microplate Washer, Thermo Fisher Scientific) when we washed the plates. We set the cutoff value as 0.5 (O.D.) according to the neutralizing activity confirmed by the neutralization assay.

**Measurement of IFN-α2 concentration**

We tested the serum IFN-α2 concentration with the ProQuantum^TM^ Human IFN alfa Immunoassay Kit (Invitrogen, MA, USA) according to its technical guide. Briefly, we diluted samples 10-fold with assay dilution buffer, mixed 5 μL of diluted samples with the same amount of antibody-conjugate mixture and incubated them for 1 hour at room temperature. After incubation, we added 40 μL of qPCR mixture to each sample and measured them with a StepOnePlus Real-Time PCR System (Applied Biosystems, MA, USA) and analyzed them with StepOne^TM^ Software. Finally, we multiplied the measured IFN-α2 value by 10 to return to the in vivo concentration.

**Statistical analysis**

We carried out statistical analysis using JMP software (SAS Institute, NC, USA). The variables that did not follow a normal distribution are expressed using the median and interquartile range (IQR). Nominal variables are expressed as numbers and percentages. To analyze the effect of dichotomous variables, the χ2 test was used with Fisher’s exact test. Correlations were estimated by the listwise method. We generated figures of the IFN-α2 concentration with GraphPad Prism 7 (GraphPad Software, CA, USA).

**References**

1. Bastard P, Gervais A, Le Voyer T, Rosain J, Philippot Q, Manry J, et al. Autoantibodies neutralizing type I IFNs are present in ~4% of uninfected individuals over 70 years old and account for ~20% of COVID-19 deaths. Sci Immunol 2021; 6.

2. Bastard P, Rosen LB, Zhang Q, Michailidis E, Hoffmann HH, Zhang Y, et al. Autoantibodies against type I IFNs in patients with life-threatening COVID-19. Science 2020; 370.
